# Supplementary material for: Mechanisms governing the pioneering and redistribution capabilities of the non-classical pioneer PU.1
Source: Nat Commun. 2020 Jan 21;11:402. doi: 10.1038/s41467-019-13960-2 (PMC6972792; doi:10.1038/s41467-019-13960-2)
Supplement: Supplementary file 7 — Source data [file 41467_2019_13960_MOESM7_ESM.zip › Source_Data/Figure6/Figure6B_MotifScanOutput/ETS1_mutPU.1/homerResults/motif10.similar.html]

motif10

## Information for motif10

A
G
T
C
G
A
C
T
A
C
G
T
C
A
G
T
T
A
C
G
G
C
T
A
C
G
A
T
A
T
C
G
C
G
A
T
C
A
T
G
C
A
T
G
A
G
C
T
  
Reverse Opposite:  

T
C
G
A
G
T
A
C
G
T
A
C
G
C
T
A
T
A
G
C
G
C
T
A
C
G
A
T
A
T
G
C
G
T
C
A
T
G
C
A
C
T
G
A
T
C
A
G
  

|  |  |
| --- | --- |
| p-value: | 1e-170 |
| log p-value: | -3.929e+02 |
| Information Content per bp: | 1.649 |
| Number of Target Sequences with motif | 746.0 |
| Percentage of Target Sequences with motif | 5.53% |
| Number of Background Sequences with motif | 356.0 |
| Percentage of Background Sequences with motif | 1.01% |
| Average Position of motif in Targets | 102.2 +/- 44.0bp |
| Average Position of motif in Background | 100.6 +/- 58.0bp |
| Strand Bias (log2 ratio + to - strand density) | 0.0 |
| Multiplicity (# of sites on avg that occur together) | 1.03 |
| Motif File: | file (matrix) reverse opposite |

### Similar de novo motifs found

|  |  |  |  |  |  |  |  |
| --- | --- | --- | --- | --- | --- | --- | --- |
| Rank | Match Score | Redundant Motif | P-value | log P-value | % of Targets | % of Background | Motif file |
| 1 | 0.869 | A G T C A G C T A C G T A C G T A T C G G C T A C G A T A T C G C G A T A C T G C A T G | 1e-154 | -355.800323 | 14.51% | 6.56% | motif file (matrix) |
| 2 | 0.873 | A G T C A G C T A C G T C G A T T A C G C G T A C G A T A T C G A C G T | 1e-149 | -343.268482 | 13.99% | 6.32% | motif file (matrix) |
| 3 | 0.788 | C G T A A T G C G C A T A C G T G T A C C G T A C T G A C G T A C T A G T C A G T G C A G T C A | 1e-105 | -243.477634 | 7.78% | 3.01% | motif file (matrix) |
| 4 | 0.788 | A G T C C A G T A G C T G C A T A T C G T C G A G C A T T A C G | 1e-103 | -238.572035 | 9.17% | 3.96% | motif file (matrix) |
| 5 | 0.892 | T G C A G T A C G T A C C G T A A G T C G A C T A C G T T A G C G C T A T C G A C T G A T C A G T G A C | 1e-91 | -211.262277 | 11.21% | 5.65% | motif file (matrix) |
| 6 | 0.763 | G C T A T G C A T G C A A G T C G A C T A C G T T A G C C G T A T C G A C T G A T C A G T G C A T G C A T G C A | 1e-60 | -139.893533 | 4.02% | 1.44% | motif file (matrix) |
